# Absence of evidence is not evidence of absence for first trimester dydrogesterone-induced birth defects

**DOI:** 10.1093/hropen/hoae030

**Published:** 2024-05-11

**Authors:** Reshma Quadros, Gaurav Puppalwar, Amey Mane, Suyog Mehta

**Affiliations:** Department of Medical Affairs and Clinical Research, Sun Pharmaceutical Industries Ltd., Mumbai, India; Department of Medical Affairs and Clinical Research, Sun Pharmaceutical Industries Ltd., Mumbai, India; Department of Medical Affairs and Clinical Research, Sun Pharmaceutical Industries Ltd., Mumbai, India; Department of Medical Affairs and Clinical Research, Sun Pharmaceutical Industries Ltd., Mumbai, India

Sir,

We greatly appreciate the meta-analysis by Katalinic *et al.* (2024) entitled ‘No additional risk of congenital anomalies after first-trimester dydrogesterone use: a systematic review and meta-analysis’. This meta-analysis has certainly initiated a discussion on safety concerns associated with the use of dydrogesterone in the first trimester of pregnancy.

We are, however, concerned by the assertion/claim in the title of this review, particularly due to the suboptimal power of this meta-analysis. Among the small number of studies (six RCTs and three observational studies) included in this review, three RCTs had a high risk of bias; two observational studies had a serious risk of bias and the third observational study had a critical risk of bias. The congenital anomalies measured by these selected studies were not the primary outcome and were not documented in a standardized way. We further express concerns on the selection criteria of this meta-analysis, which limit the true detection and causal assessment of congenital anomalies. For instance, while some congenital heart diseases may be diagnosed during pregnancy, some others are not detected until after birth or much later in life: for example, during childhood or adulthood (DeSilva *et al.*, 2016).

Nevertheless, Katalinic *et al.* have generated valuable evidence by analyzing the prevalence rate of congenital anomalies associated with dydrogesterone use. We are, however, perplexed by three additional studies (Huang *et al.*, 2019; Jiang *et al.*, 2022; Zhang *et al.*, 2022) being included in this analysis despite not meeting the eligibility criteria. Their inclusion should be justified as they may have a significant implication on the conclusion drawn from this analysis, i.e. the prevalence range is within the range published by the EUROCAT Network for congenital anomalies in Europe.

Pharmacovigilance (PV) continues to monitor the safety of a drug following its approval and marketing. This monitoring system is especially important with respect to rare adverse drug reactions (ADRs), or those that occur after long-term drug exposure in certain groups. The importance of post-marketing drug safety monitoring can be supported by the fact that one-third of safety issues identified by PV are added to the warnings and precautions section on drug pack labels (Lavertu *et al.*, 2021). A wealth of PV data, in addition to being a ‘sign giver’, may form the cornerstone in identifying some serious ADRs, which, in some cases, go unrecognized until long after the drug has been approved for the market.

An abstract by Henry *et al.* (2023) described a global PV study reporting birth defects associated with use of oral dydrogesterone in ART. Since its publication, we have closely monitored the literature for new evidence to gain some clarity on this topic. We feel that there is a chance that this abstract has escaped the attention of Katalinic *et al.* Among the 29 million case safety reports included in Henry *et al.*’s case–non case PV study, more than 50 000 were related to the use of drugs for ART (Henry *et al.*, 2023). With respect to birth defects, 60 out of the 375 cases reported were associated with dydrogesterone use (16%). Compared to any other drug, the incidence of birth defects linked to dydrogesterone use was 5.4 times higher. Furthermore, when dydrogesterone was compared to other drugs used in ART, the incidence of birth defects was 5.9 times higher. Similarly, in a head-to-head comparison with progesterone, a 5.4 times higher incidence of birth defects was associated with dydrogesterone use (Henry *et al.*, 2023).

Systematic reviews and meta-analyses represent the pinnacle of evidence with practice changing implications. Katalinic *et al.*’s (2024) meta-analysis, despite being a useful piece of evidence, is not robust enough to conclude the safety of dydrogesterone use. One would agree that absence of evidence is not the evidence of absence. We are grateful to Katalinic and colleagues for their review; however, more large-scale and robust RCTs or meta-analyses focusing on long-term outcomes are required to address the safety concerns with respect to congenital anomalies associated with dydrogesterone use in the first trimester of pregnancy.
